# Bilateral heterochronic spontaneous hemothorax caused by pulmonary arteriovenous malformation in a gravid: A case report

**DOI:** 10.1186/1749-8090-5-96

**Published:** 2010-10-31

**Authors:** Yinghao Zhao, Guang-Yu Li, Zhiguang Yang, Peng Zhang, Kun Zhang, Guoguang Shao

**Affiliations:** 1 Department of Thoracic Surgery, The Second Hospital of Jilin University, 218 Ziqiang Street, Changchun, China; 2 Department of Ophthalmology, The Second Hospital of Jilin University, 218 Ziqiang Street, Changchun, China; 3 Department of Central research laboratory, The Second Hospital of Jilin University, 265 Ziqiang Street, Changchun, China

## Abstract

Bilateral heterochronic spontaneous hemothorax as a result of pulmonary ateriovenous malformation is a very rarely happened disease. A 34-year-old woman presented major symptoms with right-sided chest pain and shortness of breath. The following contrast-enhanced computed tomographic scan of the chest showed a large amount of fluid in the right thorax with mediastinal shift, but without major vessel injury and 2 small dense opacities in the apical segment of the right lower lobe and in the posterior aspect of the left lower lobe. The patient underwent local resection of the right lower lobe. The pulmonary ateriovenous malformation was further identified by pathological examination. One month after she was discharged home, the symptoms described above recurred. A follow-up computed tomographic scan of the chest showed a large amount of fluid in the left thorax. During the emergency operation, we found a bullous lesion in the left lower lobe and a small blood vessel overlying the lesion that was actively bleeding. As stated above, local resection of the left lower lobe was performed once more. Pathological result was the same as observed previously. There were no postoperative complications and she was discharged from the hospital after two weeks. Two months later, she successfully delivered a healthy female infant. Up to now, regular follow-up observation has shown her to be perfectly asymptomatic.

## Background

Nontraumatic hemothorax is distinctly uncommon: bilateral heterochronic spontaneous hemothorax is rarer. They may result from a variety of causes, and in some patients the cause can remain unknown even after exploratory thoracotomy. We submit a case of bilateral heterochronic spontaneous massive hemothorax as a result of pulmonary ateriovenous malformation that presented during the pregnancy.

## Case presentation

This is the case of a 34-year-old female who at 22 weeks' gestation was diagnosed with spontaneous hemothorax associated with pulmonary arteriovenous malformation and treated with local resection of the right lower lobe. She presented with sudden onset of right-sided chest pain associated with shortness of breath at the emergency department of her local hospital. The pain continued for two hours without any treatment and she felt gradually increasing heaviness in the chest, which was associated with mild dyspnoea. There was no cough, expectoration, hemoptysis, wheezing, hoarseness of voice or pedal swelling. The patient was 6 months pregnant.

**Figure 1:**
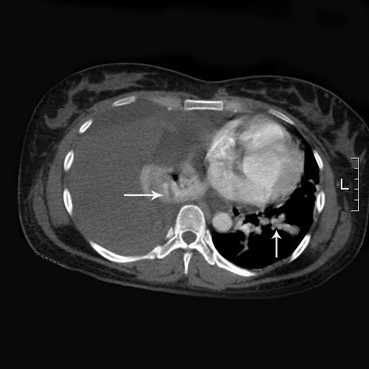
**Sample figure title**. Contrast-enhanced computed tomographic scan of the thorax shows blood in the right pleural space and compression atelectasis of the right lung. Note also the high-density nodules (arrow) located right lower lobe and left lower lobe, possibly indicative of the PAVMs.

**Figure 2:**
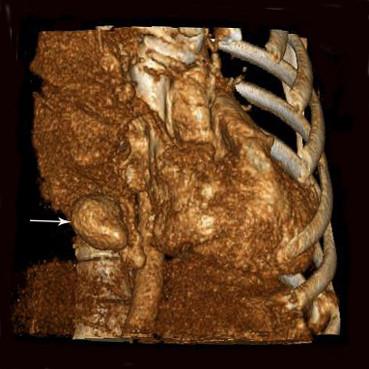
**Another sample figure title**. Three-dimensional (3-D) computed tomographic restruction of the thorax shows the high-density nodule (arrow), possibly indicative of the PAVM.

Upon examination at our institution, dullness was noted in the right thorax. An urgent electrocardiogram was normal and a routine hemogram showed hemoglobin of 11.2 gm%, with a total leukocyte count of 11500/cmm (80% neutrophils). Bleeding time, clotting time, serum fibrinogen, liver function tests and renal function tests were normal. A follow-up contrast-enhanced and three-dimensional (3-D) computed tomographic scan of the chest (Figure 1, 2) showed a large amount of fluid in the right thorax with mediastinal shift, but with no major vessel injury and 2 small dense opacities in the apical segment of the right lower lobe (diameter = 2.5 cm) and in the posterior aspect of the left lower lobe (diameter = 1.5 cm). In the absence of another cause for spontaneous hemothorax on either clinical or imaging grounds, we determined that these might represent small pulmonary arteriovenous malformations. A pleural aspiration was planned. While waiting for the operation, the patient developed severe right-sided chest pain associated with increasing shortness of breath. Her blood pressure fell to 90/60 mmHg and oxygen saturation was 92-95%.

**Figure 3:**
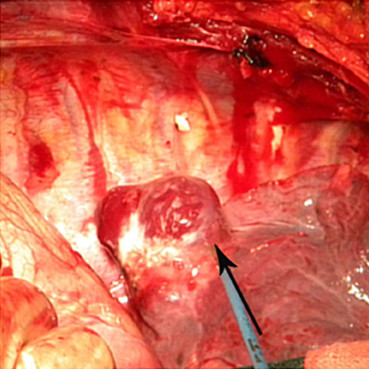
**Another sample figure title**. Intraoperative view of the active "bleeder" (arrow): the PAVM overlying a bullous lesion in the right lower lobe.

The patient was immediately taken to the operating theater, where she underwent local resection of the right lower lobe. Perioperatively, we found a bullous lesion in the right lower lobe and a small blood vessel overlying the lesion that was actively bleeding, in addition to massive blood clots (about 3000 ml) (Figure 3). These were clipped and a local resection of the right lower lobe was performed. Perioperatively, the patmient required 10 units of blood. Postoperatively, she was moved to the intensive care unit, where she was extubated after 3 hours; the next day, she was moved to the ward in a stable condition. The chest drains were removed after a couple of days. She and fetus were doing very well after the operation. Pathology revealed lung tissue in the lumens of the larger vessels, alveolar interval telangiectasia and hyperemia. The PAVM was identified. There were no postoperative complications Considering the safety to the fetus, the weakness of the patient and embolization complications such as radiation injury, we took relatively conservative treatment-observation. She was discharged from the hospital within three weeks. We also explained to the patient need to pay a close attention to the symptoms such as chest pain and dyspnea after discharge home. If she doesn't feel better, she needs to return hospital to have examination as soon as possible. If the patient's healthy situation improved, she should come back to the hospital again to treat the contralateral PAVM with pulmonary artery embolization.

One month after the patient was discharged from the hospital, the symptoms described above recurred. A follow-up computed tomographic scan of the chest showed a large amount of fluid in the left thorax. During the emergency operation, we found a bullous lesion in the left lower lobe and a small blood vessel overlying the lesion that was actively bleeding. As stated above, local resection of the left lower lobe was performed. Pathology was the same as observed previously. After two weeks in the hospital, the patient had made a complete recovery and returned to her normal routine. Two months later, she delivered a healthy female infant. Regular follow-up observation have shown her to be perfectly asymptomatic and the patient has returned to her normal routine.

## Discussion

Spontaneous hemothorax is a rare complication of pulmonary arteriovenous malformation (PAVM) which may be defined as direct communications between branches of a pulmonary artery and pulmonary vein, without an intervening pulmonary bed [1-3]. The incidence of PAVM is rare, and more than 40% of cases are singular [4,5]. The incidence of bilateral PAVM ranges from 8-20% [4,6]. Bilateral heterochronic spontaneous hemothorax is rarer. Notably, there is an association with Osler-Weber-Rendu disease [7].

Pregnant women are at a particular risk of serious complications. Pregnancy can increase the size of PAVMs, due to increased blood volume and cardiac output, to hormonal effects on the blood vessels, or both. The change in blood volume and cardiac output lead to increased pulmonary blood flow: preferential blood flow across the PAVM leads to its dilatation [8]. In addition, the increased level of progesterone can increases the venous distensibility and may cause further augmentation of blood flow through a pre-existing PAVM, leading to progression in size of PAVM [8,9].

Pulmonary arteriovenous malformations may be clinically silent or present with respiratory failure with cyanosis, exercise intolerance, polycystemia, and clubbing. Hemothorax is a rare complication of PAVM and is potentially fatal [1]. Contrast-enhanced computed tomography is a valuable and widely available diagnostic tool for patients with abnormal chest radiography with suspicious of PAVM. In pregnancy, PAVM can at first be mistaken for pulmonary embolism, which is a far more common cause of respiratory distress and reduced breath sounds upon clinical examination [1]. Hemothorax secondary to PAVM is suggested by reduced tactile vocal fremitus and by a stony dull percussion note. This diagnosis can be confirmed by hazy opacification upon chest radiography and by the appearance of fluid upon spiral computed tomography. Initial management with therapeutic embolization may well be appropriate in pregnant patients who present with symptomatic PAVM [3]. In patients who are hemodynamically unstable or in whom embolization has failed, open surgical resection of the PAVM has been successful [3].

To the best of our knowledge, our case is the only report of bilateral heterochronic spontaneous massive hemothorax in a gravida. Spontaneous hemothorax secondary to PAVM, particularly a bilateral heterochronic case, is a rare clinical entity that is life-threatening if diagnosis and intervention are delayed. Surgical resection of a PAVM is an acceptable option in those patients who present massive spontaneous hemothorax.

## Competing interests

The authors declare that they have no competing interests.

## Authors' contributions

YHZ had made contribution to design of the manuscript and had been involved in drafting and critically revising the manuscript. GGS performed the operation, had been involved in drafting the manuscript and had given the final approval to publish the manuscript. Guang-Yu Li and ZGY had been involved in critically revising the manuscript. PZ and KZ had been involved in drafting the manuscript. All authors read and approved the final manuscript.

## Consent

Written informed consent was obtained from the patient for publication of this case report and any accompanying images.
